# Ethanolic extract of *Iris songarica* rhizome attenuates methotrexate-induced liver and kidney damages in rats

**Published:** 2020

**Authors:** Hesam Moodi, Mehran Hosseini, Mohammad Reza Abedini, Mahsa Hassanzadeh-Taheri, Mohammadmehdi Hassanzadeh-Taheri

**Affiliations:** 1 *Department of Anatomical Sciences, Birjand University of Medical Sciences, Birjand, Iran*; 2 *Cellular and Molecular Research Center, Department of Anatomical Sciences, Birjand University of Medical Sciences, Birjand, Iran*; 3 *Cellular and Molecular Research Center, Department of Pharmacology, Birjand University of Medical Sciences, Birjand, Iran *; 4 *Student Research Committee, Birjand University of Medical Sciences, Birjand, Iran*

**Keywords:** Methotrexate, Hepatotoxicity, Renal injury, Lipid peroxidation, Iris plants

## Abstract

**Objective:**

The long-term sequelae of methotrexate (MTX) remain the major cause of concern for both patients and therapists. Therefore, new approaches to decrease MTX side effects are needed. The study was carried out to evaluate the effects of *Iris songarica* Schrenk (IS) rhizome extract against MTX-induced hepatic and renal injuries in rats.

**Materials and Methods:**

Forty male Wistar rats were randomly divided into five groups (n=8) including control, MTX, IS50, IS150 and IS300. Control and MTX groups were only treated orally with saline; whereas, IS50, IS150 and IS300 groups were treated with IS extract at three different doses (50, 150, and 300 mg/kg, respectively). Besides, the MTX and experimental groups were received a single dose of MTX (20 mg/kg) intraperitoneally on day 4. On the ninth day, animals were sacrificed, blood transaminases, urea and creatinine were assessed and the concentration of malondialdehyde (MDA) and the activity of super-oxide dismutase (SOD) were determined in both liver and kidney tissues. Moreover, hepatic and renal damages were evaluated histopathologically.

**Results:**

MTX by increasing oxidative stress (MDA) and decreasing antioxidant capacity (SOD) induced hepatic and renal damages as confirmed by biochemical and histological parameters analyses. However, treatment with IS caused significant improvements in hepatic and renal histological architectures and SOD activity (p<0.01) along with reducing liver enzymes, urea, creatinine and MDA (p<0.01).

**Conclusion:**

The results of the present study showed that IS extract through antioxidant and probably anti-inflammatory activities, could effectively limit MTX-induced hepatic and renal injuries in rats.

## Introduction

Since 1956, when it was first reported that methotrexate (MTX), as a folate antagonist, successfully treated LI210 leukemia-bearing mice and introduced as an anti-cancer drug to the clinic, the spectrum of its therapeutic applications has expanded (Cronstein and Bertino, 2000). Though MTX was first developed to treat malignancies, it is now clinically applied for a wide range of illnesses such as gynecological complaints, inflammatory arthritis, skin diseases and possibly other disorders (Maestá et al., 2018 ▶; Shah et al., 2016 ▶; Weinblatt, 2018 ▶). MTX is the mainstay of treatment in rheumatoid arthritis (RA) (Becciolini et al., 2016 ▶).

Despite the fact that MTX holds a unique place in the treatment of different disorders like RA, it has some disadvantages such as hepatotoxicity and nephrotoxicity (Asci et al., 2017 ▶; Taylor et al., 2019 ▶). The drawbacks and long-term complications of MTX stay the fundamental causes of disquiet for both patients and therapists. Therefore, new approaches to improve patients' tolerance and decrease MTX side effects are urgently needed (Nurgali et al., 2018 ▶). Subsequently, this issue has generated a lot of interest and many studies are being done in this area. A number of natural products are under investigation or were experimentally tested to determine their potential preventive effects (Bu et al., 2018 ▶;Kalantari et al., 2019 ▶; Mehrzadi et al., 2018 ▶). Use of natural products with anti-inflammatory potential could be a promising approach because they may have synergistic effects with MTX and consequently, by reducing the required dose of MTX might reduce its side effects. Moreover, they can protect the liver and kidney from devastating effects of MTX such as oxidative stress, inflammation, and fibrogenesis by their inherent potentials (De et al., 2018 ▶;Khafaga and El-Sayed, 2018 ▶). 

**Figure 1:**
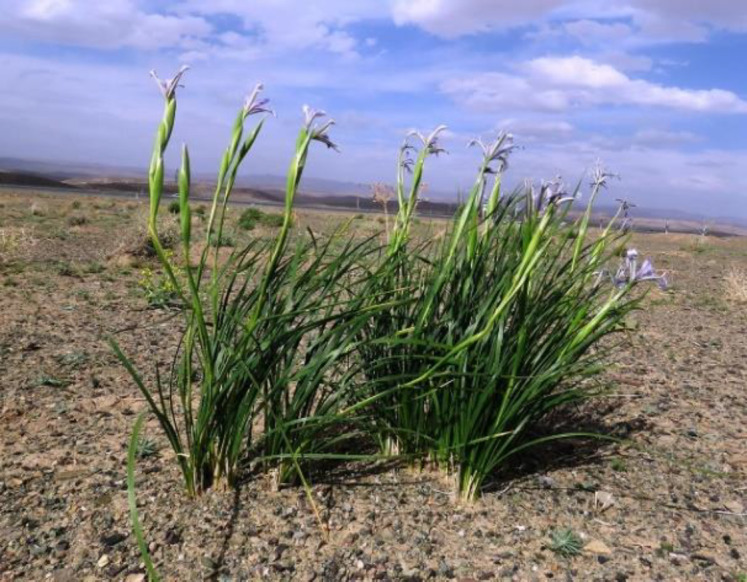
*Iris songarica* Schrenk in the flowering stage (April 2018), Mood, Birjand, Iran (37.71ºN, 59.49ºE)

We previously reported that ethanolic extract of rhizome of* Iris songarica* Schrenk exhibited a profound anti-inflammatory and antinociceptive potentials in mice (Ahani et al., 2017 ▶). *Iris songarica* S. (IS) is a rhizomatous herbaceous perennial widely distributed in Iran (Figure 1), Afghanistan, Pakistan, Kazakhstan, Tajikistan, Turkmenistan, Uzbekistan and North China (King and Killens, 2012 ▶). The genus *Iris* includes more than 300 species and its medicinal plants are known as Irsa in Iranian traditional medicine. Interestingly, according to a famous book of traditional Iranian medicine, The Canon of Medicine, Irsa has been prescribed for liver and spleen disorders (Pour et al., 2019 ▶). However, to the best of our knowledge, no study has yet investigated IS in this aspect. Therefore, we designed the present study to evaluate the effects of IS rhizome extract against MTX-induced hepatic and renal injuries. On this ground, biochemical markers in plasma representing liver and kidney functions, tissue oxidative and antioxidant markers, and histological assessments of hepatic and renal tissues were considered.

## Materials and Methods

**Plant material and extract preparation**

The plant *Iris songarica* Schrenk (including rhizome, leaves, and stem) was collected from Mood (37.71ºN, 59.49ºE), Birjand, Iran. The plant is not endangered or protected species in Iran. Its taxonomy was confirmed by an expert botanist (Department of agriculture, University of Birjand, Iran), and a voucher specimen (HR: 520) was deposited at the herbarium of agriculture school, University of Birjand, Birjand, Iran. 

The rhizomes of IS were cleaned, dried in shade, powdered, and then, stored in a cool and dry place until use. To prepare the ethanolic extract, 100 g of the powdered rhizome was macerated in 80% ethanol (900 ml) with constant stirring at room temperature (25ºC) for 24hr followed by filtration through a Whatman filter paper (Grade 589, Blue Ribbon, Germany). Afterwards, the extract was concentrated by a rotary evaporator (at 35ºC) and freeze-dried (Hassanzadeh-Taheri et al., 2016 ▶). The yield of extraction was around 4.8%. 

**Animals**

All the experimental procedures done in the present study, were in accordance with the guidelines for animal care and use, and approved by the Research Ethics Committee of Birjand University of Medical Sciences, Iran (Permit code: IR.BUMS.REC.1398.023). All efforts were made to minimize animals suffering and reduce the number of animals used.

 Forty male Wistar rats, weighing 200–230 g (9weeks old), were kept in a temperature-controlled room (20-24°C) with 12:12 hr light/dark cycle. All rats had free access to the standard commercial laboratory animal's diet (Behparvar Co, Karaj, Iran) and tap water. The animals were randomly allocated into five groups each containing eight rats, as follows: group 1 (control) and group 2 (MTX) were treated with saline, group 3 (IS50) was treated with 50 mg/kg IS extract, group 4 (IS150) was treated with 150 mg/kg IS extract, and group5 (IS300) was treated with 300 mg/kg IS extract. All treatments were administrated orally once daily for 8 consecutive days. The MTX and the experimental groups (IS50, IS150, and IS300) received a single intraperitoneal (i.p.) injection of MTX (20 mg/kg, EBEWE Pharma, Austria) on day 4 (Bu et al., 2018 ▶). Since in our previous study, 50-300 mg/kg IS extract doses were found effective to control pain and inflammation (Ahani et al., 2017 ▶), these doses were also used in the present study.

On the ninth day and 24 hr after the last administration, all animals were anesthetized by i.p. injection of 65 mg/kg ketamine (Rotexmedica, Germany) and 10 mg/kg xylazine (Alfasan, Netherland) (Hassanzadeh-Taheri et al., 2018a ▶). Blood samples were collected from the heart, centrifuged (at 3000 g for 10 min), and the obtained plasma was kept (-60ºC) for further analyses. Immediately after blood collection, animals' kidneys and livers were dissected and weighed. One portion of each organ was used to prepare tissue homogenate (tissue: phosphate buffered saline, 1:9 w/v) whereas, another portion was fixed in 4% paraformaldehyde solution for histological assessments.

**Biochemical assessment**

The biochemical parameters including aspartate aminotransferase (AST), alanine aminotransferase (ALT), urea and creatinine (Cr) were determined using standard kits (Bionik, Iran) and an auto-analyzer system (Prestige 24i-Japan).

**Assessment of protein concentrations and superoxide dismutase (SOD) activity**

A sample of each liver/kidney tissue (100 mg) was homogenized in 900 µl of cold phosphate buffered saline (PBS, pH 7.4), and centrifuged at 5000g for 15 min and protein concentration was determined in the supernatants using Bradford's assay. Accordingly, 20 µl supernatant was dissolved in200 ml Coomassie brilliant blue reagent (G250), then, incubated at room temperature for 10 min and the absorbance was read at 595 nm. The standard curve was plotted using different concentrations (10-100 µg/ml) of bovine serum albumin solutions. 

The SOD activity was evaluated according to the reduction rate of WST-1 [4-(3- (4-iodophenyl)-2-(4-nitrophenyl)-2H-5-tetrazolio)-1, 3-benzene disulfonate]. Briefly, 50 µl supernatant was transferred into each well and then, 250 µl of reaction mixture [containing 45 µl of assay buffer (50 mM Na_3_PO_4_, 0.1 mM diethylenetriamine pentaacetic acid and 0.1 mM hypoxanthine in 20 ml) with 100 μl of 10 mM WST-1 solution, 100 μl of 2 mg/ml catalase and 5 μl of xanthine oxidase (at the final concentration of 4.5 mU/ml) ] was added to the well and mixed appropriately. After 5 min incubation at room temperature, the absorbance was read at 405 nm and the SOD activity was expressed as U/mg protein (Zhang et al., 2016 ▶). 

**Assessment of lipid peroxidation**

Lipid peroxidation in the liver/kidney homogenate was determined by measuring the amounts of malondialdehyde (MDA), the end-product of lipid peroxidation process. MDA reacts with thiobarbituric acid (TBA) as a thiobarbituric acid reactive substances (TBARS) to form a 1:2 MDA-TBA adduct, which has a strong absorption at 532 nm. Briefly, 100µl supernatant of liver/kidney homogenate was added to 600 µl orthophosphoric acid (1%) and 200 µl of TBA (0.67%). The mixture was heated at 90°C for 45 min, the reaction was stopped by placing samples on ice; then, 800 µl N-butanol was added, vortexed and centrifuged for 20 min at 5000 g. The resulting supernatant was removed and its optical absorbance was measured at 532 nm. Finally, MDA concentration was expressed as nmol/mg protein (Ataie et al., 2019 ▶).

**Histological assessment**

Liver and kidney tissues were processed by conventional histological methods and paraffin blocks were prepared. Then, tissue sections with a thickness of five micrometers, were prepared using a microtome. Slides were stained with hematoxylin and eosin dyes. For each rat, three random slides (9 sections, 90 unbiased fields) were examined using a light microscope (UPLAN FI, Japan) to study MTX toxicity associated with histological alterations in the studied tissues. Pathological lesions including degeneration, congestion, infiltration, and hemorrhage were quantified according to a scoring checklist (0=none, 1=mild, 2=moderate, and 3=severe)(Hassanzadeh-Taheri et al., 2018b ▶). Finally, the mean scores were compared among the studied groups. 

**Statistical Analysis**

Results are presented as mean ± SD in all groups. The homogeneity of data variance was checked by the Shapiro–Wilk test. The groups were compared using ANOVA and Dunnett's T3 *post hoc* tests. Furthermore, histopathological grading scores were analyzed among the groups using Kruskal-Wallis and Mann-Whitney U tests. Statistical analysis was performed using SPSS 22 (SPSS Inc. Chicago, IL, USA) software. P-values less than 0.05 were considered significant. 

## Results

**Biochemical findings**

To evaluate the overall performance of the liver and kidney, biochemical parameters indicative of their functions including plasma concentrations of AST, ALT, urea and Cr were assessed (Table 1). The results of the liver enzymes assessment showed that the levels of AST and ALT markedly increased (p<0.001 and p<0.01 respectively) in the MTX group compared to the control group. However, treatment of MTX-injected rats with IS extract (50-300 mg/kg b.w.) significantly ameliorated the AST and ALT elevations. Treatment with IS extract at the dose of 50 mg/kg, restored plasma concentration of AST to normal level while in the IS150 and IS300 groups, AST levels were still significantly higher than normal values (p<0.05 and p<0.01, respectively). Similar to AST, the treatment of rats with the IS extract (50-300 mg/kg) significantly reduced the ALT level compared to the MTX group (p<0.001 for all). Surprisingly, treatment with IS extract (irrespective of the doses) not only statistically decreased ALT levels compared with MTX group, but also at all doses reduced its levels even significantly lower than the control group values (p<0.001 all). 

The results of renal function tests revealed that MTX injection elevated plasma urea and Cr in rats. The concentration of blood urea in the MTX group was significantly (p<0.001) higher than control rats. Upon treatment of MTX-injected rats with IS extract (50-300 mg/kg b.w), urea levels were markedly decreased when compared with the MTX group (p<0.001 all). Although IS extract at doses of 150 and 300 mg/kg could restore blood urea to the normal level, at the lowest dose (50 mg/kg), blood urea concentration was still higher than control group (p<0.05). Similar to urea, MTX-injected rats exhibited a significant increase in the plasma concentration of Cr in comparison to normal rats (p<0.01). Consistent with previous findings, IS extract treatment (50-300 mg/kg) reduced Cr levels compared to the MTX group (p<0.001 for all) and restored the levels to normal values at all doses.

**Hepatic and renal weight changes**

The liver or kidney indexes (liver or kidney weight to body weight) in different studied groups are shown in Table 2. The liver index was significantly (p<0.01) increased in the MTX group compared to the control, and none of the doses of IS could alleviate this elevation. Assessment of kidney index did not reveal any statistically significant difference among groups.

**Table 1:** Effect of ethanolic extract of *Iris songarica* (IS) on the plasma levels of AST, ALT, urea and creatinine

**Groups**	**AST (U/L)**	**ALT (U/L)**	**Urea (mg/dl)**	**Creatinine (mg/dl)**
**Control**	61.12±3.27^###^	59.00±4.59^##^	39.62±3.29^###^	0.68±0.06^#^
**MTX**	116.00±12.03^***^	77.25±7.70^**^	55.62±3.15^***^	0.82±0.07 ^*^
**MTX+IS50**	71.75±9.05^###^	42.12±3.31^***,###,&^	46.37±6.23^#^	0.65±0.05 ^##^
**MTX+IS150**	69.12±5.11^###,*^	41.12±5.30^***,###^	37.71±4.71^###^	0.61±0.06 ^##^
**MTX+IS300**	73.62±6.04^**,###^	35.62±4.13^***,###,^^†^	38.37±3.42 ^###^	0.65±0.07 ^##^

Values are presented as mean ± SD (n=8). Data were analyzed by one-way ANOVA test followed by Dunnett's test. *p<0.05, **p<0.01 and ***p<0.001, show significant difference compared to control group. # p<0.05, ## p<0.01, and ### p<0.001, show significant difference compared to MTX group.^ &^p<0.05, shows significant difference compared to IS300 group. †p<0.05, shows significant difference compared to IS50 group. MTX: methotrexate (20 mg/kg), IS50-IS300: ethanolic extract of *Iris songarica* at the doses of 50-300 mg/kg. AST: aspartate aminotransferase, ALT: alanine aminotransferase.

**Table 2:** Effect of ethanolic extract of *Iris songarica* (IS) on hepatic and renal MDA and SOD

**Groups**	**Organ index** (g/g body weight)*100		**MDA (nmol/mg protein)**		**SOD (U/mg protein)**	
	**Liver**	**Kidney**	**Liver**	**Kidney**	**Liver**	**Kidney**
**Control**	2.56±0.15^##^	0.38±0.02	1.98±0.107^###^	1.69±0.09^###^	2.96±0.13^###^	1.87±0.12^###^
**MTX**	3.13 ±0.16^**^	0.35±0.04	3.11±0.18^***^	4.79±0.28^***^	1.66±0.12^***^	1.55±0.11^***^
**MTX+IS50**	3.24±0.32	0.38±0.04	1.95±0.05^###,&,†^	2.85±0.106^###,†,&^	1.75±0.11^***,###^	1.83±0.12^***,###^
**MTX+IS150**	3.40±0.17^***^	0.37±0.02	1.66±0.09^***,###,@,†^	2.46±0.09^***,###,@,&^	1.75±0.12^***,###^	1.78±0.11^***,###^
**MTX+IS300**	3.33±0.26^***^	0.40±0.04	1.20±0.16^***,###,&,†^	2.31±0.22^***,###,@^	1.75±0.11^***,###^	1.69±0.11^***,###^

Values are presented as mean±SD (n=8). Data were analyzed by one-way ANOVA test followed by Dunnett's test. *p<0.05, **p<0.01 and ***p<0.001, show significant difference compared to control group. # p<0.05, ## p<0.01, and ### p<0.001, show significant difference compared to MTX group respectively.^@, †^ and ^&^ show significant difference at level of p<0.001 compared to IS50, IS150 and IS300 groups respectively. MTX: methotrexate (20 mg/kg), IS50-IS300: ethanolic extract of *Iris songarica* at the doses of 50-300 mg/kg. MDA: malondialdehyde, SOD: superoxide dismutase.

**Hepatic and renal tissues oxidative stress and antioxidant status**

MDA levels as a marker of oxidative stress, were assessed in hepatic and renal tissues; whereas, SOD activity was evaluated as an indicator of antioxidant defense system. The results are presented in Table 2.

In MTX-injected rats, MDA levels were significantly increased in both the liver and kidney compared with the control animals (p<0.001 both). Compared to the MTX group, administration of IS extract (all doses) significantly ameliorated the elevation of MDA concentrations in both hepatic and renal tissues (p<0.001). Compared to the control group, hepatic MDA level was restored to control level only in IS50 group. The hepatic MDA levels in IS150 and IS300 groups were significantly lower than that of the control group (p<0.01). 

Administration of MTX significantly decreased the activity of SOD in both liver and kidney tissues compared to the control group (p<0.001). Treatment with IS extract (all doses) significantly improved SOD activity in both renal (p<0.001) and hepatic (p<0.001) tissues in comparison with MTX group. However, renal and hepatic SOD levels were still lower than normal values in the all IS treated groups.

**Histological findings**

**Figure 2:**
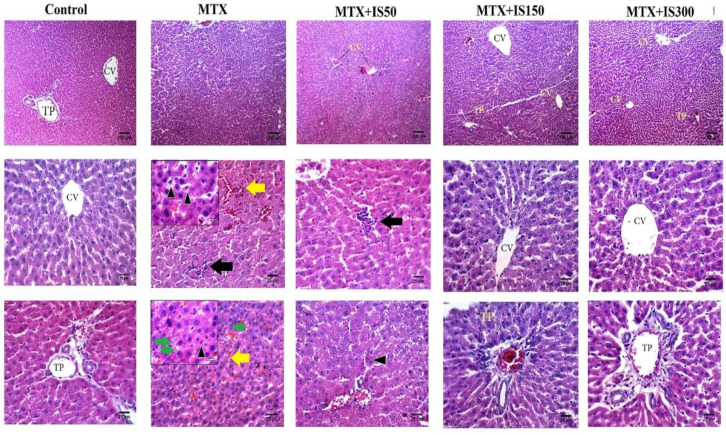
Histological examination of liver sections of control, methotrexate group (MTX), and MTX-injected rats treated with 50-300 mg/kg ethanolic extract of *Iris songarica* (IS50, IS150, and IS300). Each column belongs to a group. The top row displays 100X (scale bar=100µm) magnification; whereas, the middle and the lower rows show 400X magnification (scale bar=25µm).TP: triad port, CV: central vein, yellow arrows mark hemorrhage, black arrows indicate mononuclear cells infiltration, green arrows show microvesicular steatosis and the black arrow heads indicate pyknotic nucleus

To determine MTX and IS treatment effects on liver and kidney morphology, the tissues samples were evaluated histologically. Liver tissue sections of the control group showed normal hepatocytes radially arranged around central veins, and the portal triads did not show inflammation or congestion. On the other hand, liver tissue samples from the MTX group exhibited several histological alterations including cytoplasmic hypereosinophilia and vacuolar degenerations, necrosis around the central veins, sinusoidal dilatation and hemorrhage, mononuclear cell infiltration and congestion (Figure2). 

**Figure 3:**
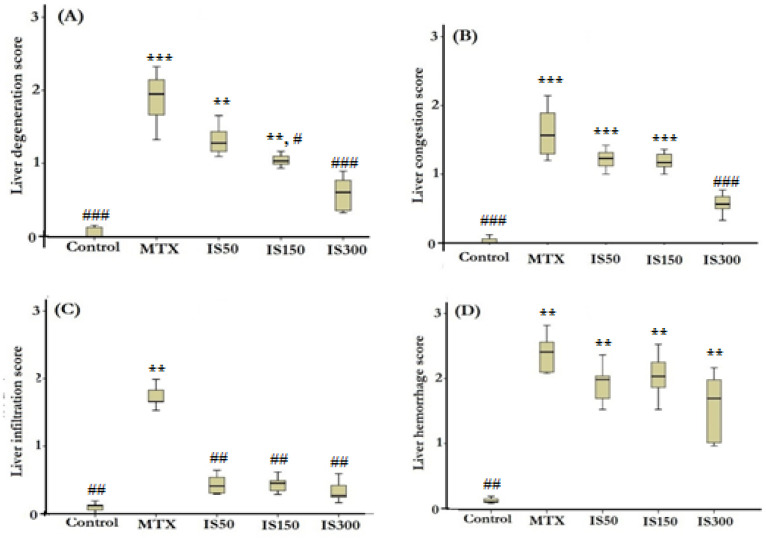
Boxplots show histological scores of liver degeneration (A), congestion (B), infiltration (C) and hemorrhage (D). MTX: methotrexate (20 mg/kg), IS50-IS300: ethanolic extract of *Iris songarica* at doses of 50-300 mg/kg. Scoring was done as none (0), low (1), mild (2) and severe (3). ^**^p<0.01 and^ ***^p<0.001, show significant difference compared to control group. ^#^p<0.05, ^##^p<0.01 and ^###^ p<0.001, show significant difference compared to MTX group

In order to make a better comparison, the alterations were scored in all microscopic slides in a blind fashion. The results of scoring comparison are presented in Figure 3. 

Accordingly, the mean rank of liver histological alterations including degeneration (p<0.001), infiltration (p<0.01), hemorrhage (p<0.01) and congestion (p<0.001) were significantly higher than those of the control group. Treatment with IS extract (all doses) could normalize infiltration while had no effect on hemorrhage. Congestion score only decreased in IS300 group to a level that was not statistically different from the control levels. Degenerative alterations significantly ameliorated in both IS150 and IS300 groups compared with MTX group, but the IS extract only at 300 mg/kg, exhibited liver histological morphology without any degenerative change.

**Figure 4:**
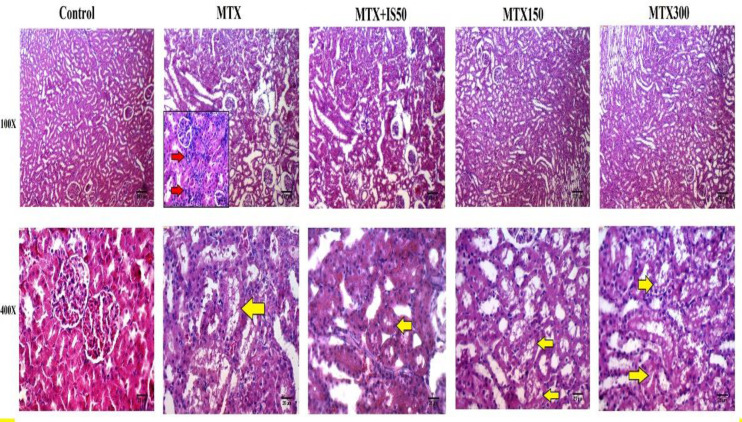
Histological examination of kidney sections of control, methotrexate group (MTX), and MTX-injected rats treated with 50-300 mg/kg ethanolic extract of *Iris songarica* (IS50, IS150, and IS300). Each column belongs to a group. The top row displays 100X (scale bar=100µm) magnification; whereas, the lower row shows 400X magnification (scale bar=25µm). The red arrows indicate mononuclear cells infiltration, and yellow arrows show tubular defects mainly vacuolated tubular epithelium

**Figure 5:**
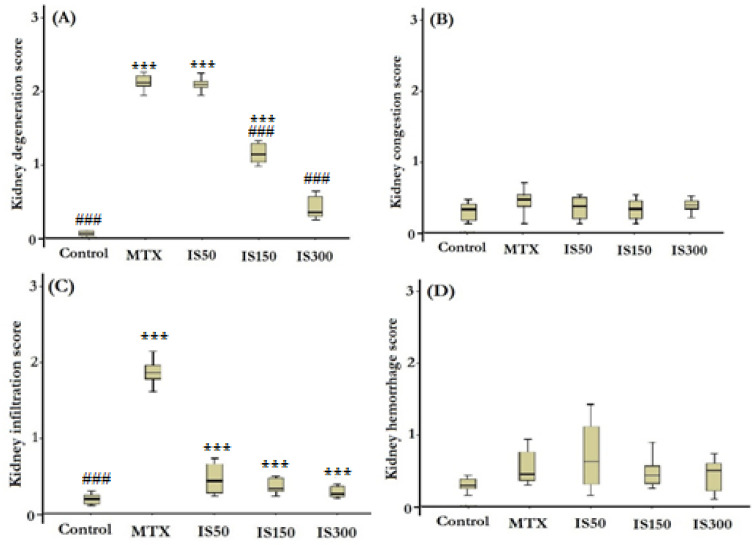
Boxplots show histological scores of kidney degeneration (A), congestion (B), infiltration (C) and hemorrhage (D) in different studied groups. MTX: methotrexate (20 mg/kg), IS50-IS300: ethanolic extract of *Iris songarica* at doses of 50-300 mg/kg. Scoring was done as follows: none (0), low (1), mild (2) and severe (3). Data were analyzed by Kruskal- Wallis test followed by Mann Whitney u test. ^***^ and ^### ^show significant difference at p<0.001 compared to control and MTX groups respectively

 The histopathological examination of kidney sections of the control group showed normal structure of glomeruli and tubules. However, histological evaluation of the kidneys of the MTX-injected rats showed different morphological degenerations including tubular defects and mononuclear cells infiltration (Figure 4, column 2). Most of tubules of MTX-injected rats were vacuolated and intracellular casts were evidently observed in their lumens (Figure 4, column 2, the yellow arrow). Histological grading scores of kidney sections revealed that degenerative changes (tubular defects) and infiltrations were markedly higher in MTX group than the control group (p<0.001 for both). Similar to the liver, IS treatment at all doses could alleviate and normalize infiltration scores in MTX-injected rats. The score of degenerative changes in renal tissues was significantly lower in IS150 and IS300 group compared to the MTX group. However, in degenerative changes scores only renal structure of IS300 group showed approximately similar morphology to the control group (Figure 5).

## Discussion

This study provides evidence on the protective role of ethanolic extract of *Iris songarica* S. (IS) against MTX-induced hepatic and renal injuries in rats. Our results showed that the liver and kidney damages were obvious in MTX-injected rats, suggesting that the model of hepatic and renal injuries was successfully established. Results of the current investigation demonstrated that a single MTX injection (20 mg/kg, i.p.) resulted in significant hepatotoxicity and renal dysfunction evidenced by increased plasma levels of AST, ALT, urea and Cr, as previously reported (Asci et al., 2017 ▶; Moghadam et al., 2015 ▶). In addition, MTX injection significantly elevated MDA level in rats’ liver and kidney, which is also consistent with the findings of previous studies (Heidari et al., 2018 ▶; Mehrzadi et al., 2018 ▶). Moreover, SOD was significantly decreased in both liver and kidney tissues which is in good agreement with the previous reports (El-Twab et al., 2019 ▶; Heidari et al., 2018 ▶). In light of the above-mentioned observations reflecting the adverse effects of MTX in the liver and kidney, it was expected to investigate liver and kidney tissues histopathologically. Prominent histological alterations in the liver tissues of MTX-injected rats including infiltration of mononuclear cells, red blood cell congestion, vacuolar degeneration and fat droplets deposition and pyknosis were observed which are in accordance with previous findings of the experimental rats exposed to MTX (Khafaga and El-Sayed, 2018 ▶; Moghadam et al., 2015 ▶). Like the liver, histopathological assessment of kidney showed serious tubular degenerations (focal tubular necrosis and presence of proteinaceous materials inside tubules), inflammatory cell infiltrations and hemorrhage. In support of our findings, previous studies demonstrated that MTX can cause marked degenerative changes mainly at tubular level. Kidney's tubules are more prone to MTX-related devastating effects due to the MTX propensity to solidify in the renal tubules in one hand and the general vulnerability of the proximal tubules due to their role in the metabolism of MTX on the other (Abdel-Daim et al., 2017 ▶; El-Twab et al., 2019 ▶). Moreover, gross examination of liver revealed that the relative weight of the liver to body significantly increased in the MTX-injected rats which is in line with the previous studies (Moghadam et al., 2015 ▶). 

Treatment of MTX-injected rats with the IS extract (all doses) could significantly reduce their AST level, but only in the group receiving the lowest dose of the IS (50mg/kg), the AST level returned to normal values.

To our knowledge, there is no study about the effects of IS on liver enzymes activity. However, in a study conducted by Akhter et al., hepatoprotective effect of *Iris spuria* rhizome methanolic extract in paracetamol-induced liver toxicity showed that 7-day oral administration of the extract at doses 100-200 mg/kg could significantly decrease liver enzyme, and hepatic MDA and increase hepatic glutathione (Akther et al., 2014 ▶). These results are in good agreement with our findings. *Iris spuria* is a species of the genus *Iris* that has similar phytochemicals (5,7-dihydroxy-2′,6-dimethoxyisoflavone) to *Iris songarica *(Akther et al., 2014 ▶; Moein et al., 2008 ▶). 

ALT is a more specific indicator of liver damage particularly in liver inflammation compared to AST (Xu et al., 2015 ▶). In the present study, MTX affected AST (1.8 fold increase) more than ALT (1.3 fold) which is similar to previous studies (Ozogula et al., 2013 ▶). Therefore, we can put forward these hypotheses that IS treatment specifically affects the liver, and all the used doses were higher than the minimum effective dose. 

IS treatment could restore plasma Cr. and urea in the MTX-injected rats. Histopathological assessment of the kidney also revealed that IS treatment reduced mononuclear cell infiltration and tubular degenerative changes which might be attributed to its anti-inflammatory and anti-oxidant properties (Ahani et al., 2017 ▶; Ayatollahi et al., 2004 ▶; Moein et al., 2008 ▶). Phytochemical assessments of IS have shown a variety of compounds such as flavonoids, isoflavonoids, glycosides, benzoquinone and triterpenoids (Iridals) (Ayatollahi et al., 2004 ▶). In addition to the mentioned phytochemicals, Ayatollahi and colleagues could isolate two specific isoflavonoids Irilin A and Irilin B from the ethanolic extract of rhizome of IS using a spectrophotometer and column chromatography (Ayatollahi et al., 2004 ▶). Four years later, they isolated seven more flavonoids from the rhizome of IS, among which Irisoid A, Irilin B and Songaricol showed antioxidant properties in HL-60 cells (with IC50 values of 21, 11 and 3.8 μg/ml, respectively) (Moein et al., 2008 ▶). 

IS treatment could significantly prevent MDA elevation and enhanced SOD activity in both liver and kidney of MTX-injected rats. IS probably could ameliorate MDA because of its ability to scavenge free radicals byirisoid A , irilin B and songaricol as previously reported (Moein et al., 2008 ▶). 

A number of medicinal plants active ingredients and crude herbal extracts were investigated to test their possible protective potential against MTX- induced renal and hepatic side effects. Rhein, chicoric acid, inulin, turmeric, gallic acid, disomin, berberin and other plant extracts were examined as an approach inhibiting MTX toxicity through antioxidant and anti-inflammatory pathways (Abdel-Daim et al., 2017 ▶; Asci et al., 2017 ▶; Bu et al., 2018 ▶; El-Twab et al., 2019 ▶; Kalantari et al., 2019 ▶; Khafaga and El-Sayed, 2018 ▶; Mehrzadi et al., 2018 ▶; Moghadam et al., 2015 ▶). 

The results of the present study provide evidence showing that *Iris songarica* rhizome extract -which is prescribed in the Iranian traditional medicine system for hepatic ailments- through antioxidant and anti-inflammatory activities, could effectively limit MTX-induced hepatic and renal injuries in rats. Last but not least, further in-depth studies on other probable biological effects of this plant would be helpful to improve our evidence-based knowledge regarding the application of this medicinal herb. 

## References

[B1] Abdel-Daim MM, Khalifa HA, Abushouk AI, Dkhil MA, Al-Quraishy SA (2017). Diosmin attenuates methotrexate-induced hepatic, renal, and cardiac injury: a biochemical and histopathological study in mice. Oxid Med Cell Longev.

[B2] Ahani A, Hassanzadeh-Taheri M, Hosseini M, Hassanpour-Fard M (2017). Antinociceptive and anti-inflammatory activities of Iris songarica schrenk rhizome ethanolic extract in mice. J Arak Uni Med Sci.

[B3] Akther N, Andrabi K, Nissar A, Ganaie S, Chandan BK, Gupta AP, Khuswant M, Sultana S, Shawl AS (2014). Hepatoprotective activity of LC–ESI-MS standardized Iris spuria rhizome extract on its main bioactive constituents. Phytomedicine.

[B4] Asci H, Ozmen O, Ellidag HY, Aydin B, Bas E, Yilmaz N (2017). The impact of gallic acid on the methotrexate-induced kidney damage in rats. J Food Drug Anal.

[B5] Ataie Z, Mehrani H, Ghasemi A, Farrokhfall K (2019). Cinnamaldehyde has beneficial effects against oxidative stress and nitric oxide metabolites in the brain of aged rats fed with long-term, high-fat diet. J Funct Foods.

[B6] Ayatollahi SA, Moein MR, Kobarfard F, Choudhary MI (2004). Two isoflavones from Iris songarica Schrenk. Daru.

[B7] Becciolini A, Biggioggero M, Favalli EG (2016). The role of methotrexate as combination therapy with etanercept in rheumatoid arthritis: Retrospective analysis of a local registry. J Int Med Res.

[B8] Bu T, Wang C, Meng Q, Huo X, Sun H, Sun P, Zheng S, Ma X, Liu Z, Liu K (2018). Hepatoprotective effect of rhein against methotrexate-induced liver toxicity. Eur J Pharmacol.

[B9] Bertino JR, Cronstein BN, Bertino JR (2000). Methotrexate: historical aspects. Methotrexate.

[B10] De S, Kundu S, Chatterjee U, Chattopadhyay S, Chatterjee M (2018). Allylpyrocatechol attenuates methotrexate-induced hepatotoxicity in a collagen-induced model of arthritis. Free Radic Res.

[B11] El-Twab SMA, Hussein OE, Hozayen WG, Bin-Jumah M, Mahmoud AM (2019). Chicoric acid prevents methotrexate-induced kidney injury by suppressing NF-κB/NLRP3 inflammasome activation and up-regulating Nrf2/ARE/HO-1 signaling. Inflamm Res.

[B12] Hassanzadeh-Taheri M, Hassanpour-Fard M, Doostabadi M, Moodi H, Vazifeshenas-Darmiyan K, Hosseini M (2018a). Co-administration effects of aqueous extract of turnip leaf and metformin in diabetic rats. J Tradit Complement Med.

[B13] Hassanzadeh-Taheri M, Hosseini M, Hassanpour-Fard M, Ghiravani Z, Vazifeshenas-Darmiyan K, Yousefi S, Ezi S (2016). Effect of turnip leaf and root extracts on renal function in diabetic rats. Orient Pharm Exp Med.

[B14] Hassanzadeh-Taheri M, Hosseini M, Salimi M, Moodi H, Dorranipour D (2018b). Acute and sub-acute oral toxicity evaluation of Astragalus hamosus seedpod ethanolic extract in Wistar rats. Pharmaceutical Sciences.

[B15] Heidari R, Ahmadi A, Mohammadi H, Ommati MM, Azarpira N, Niknahad H (2018). Mitochondrial dysfunction and oxidative stress are involved in the mechanism of methotrexate-induced renal injury and electrolytes imbalance. Biomed Pharmacother.

[B16] Kalantari H, Asadmasjedi N, Reza Abyaz M, Mahdavinia M, Mohammadtaghvaei N (2019). Protective effect of inulin on methotrexate-induced liver toxicity in mice. Biomed Pharmacother.

[B17] Khafaga AF, El-Sayed YS (2018). Spirulina ameliorates methotrexate hepatotoxicity via antioxidant, immune stimulation, and proinflammatory cytokines and apoptotic proteins modulation. Life Sci.

[B18] King C, Killens WR (2012). A guide to species irises: their identification and cultivation.

[B19] Kurutas EB (2016). The importance of antioxidants which play the role in cellular response against oxidative/nitrosative stress: current state. Nutr J.

[B20] Maestá I, Nitecki R, Horowitz NS, Goldstein DP, de Freitas Segalla Moreira M, Elias KM, Berkowitz RS (2018). Effectiveness and toxicity of first-line methotrexate chemotherapy in low-risk postmolar gestational trophoblastic neoplasia: The New England Trophoblastic Disease Center experience. Gynecol Oncol.

[B21] Mehrzadi S, Fatemi I, Esmaeilizadeh M, Ghaznavi H, Kalantar H, Goudarzi M (2018). Hepatoprotective effect of berberine against methotrexate induced liver toxicity in rats. Biomed Pharmacother.

[B22] Moein MR, Khan SI, Ali Z, Ayatollahi SA, Kobarfard F, Nasim S, Choudhary MI, Khan IA (2008). Flavonoids from Iris songarica and their antioxidant and estrogenic activity. Planta med.

[B23] Moghadam AR, Tutunchi S, Namvaran-Abbas-Abad A, Yazdi M, Bonyadi F, Mohajeri D, Mazani M, Marzban H, Łos MJ, Ghavami S (2015). Pre-administration of turmeric prevents methotrexate-induced liver toxicity and oxidative stress. BMC Complement Altern Med.

[B24] Nurgali K, Jagoe RT, Abalo R (2018). Adverse effects of cancer chemotherapy: Anything new to improve tolerance and reduce sequelae?. Front Pharmacol.

[B25] Ozogula B, Kisaoglua A, Turanb MI, Altunerc D, Senerd E, Cetine N, Ozturke C (2013). The effect of mirtazapine on methotrexate-induced toxicity in rat liver. Sci Asia.

[B26] Pour MG, Mirazi N, Seif A (2019). Treatment of liver and spleen illnesses by herbs: Recommendations of Avicenna’s heritage" Canon of Medicine". Avicenna J Phytomed.

[B27] Shah VV, Lin EJ, Reddy SP, Wu JJ (2016). Chapter 4 - Methotrexate. Therapy for Severe Psoriasis.

[B28] Taylor P, Balsa Criado A, Mongey A-B, Avouac J, Marotte H, Mueller RB (2019). How to get the most from methotrexate (MTX) treatment for your rheumatoid arthritis patient? MTX in the treat-to-target strategy. J Clin Med.

[B29] Weinblatt ME (2018). Methotrexate: who would have predicted its importance in rheumatoid arthritis?. Arthritis Res Ther.

[B30] Xu Q, Higgins T, Cembrowski GS (2015). Limiting the testing of AST: a diagnosticallynonspecific enzyme. Am J Clin Pathol.

[B31] Zhang J, Chen R, Yu Z, Xue L (2016). Superoxide dismutase (SOD) and catalase (CAT) activity assay protocols for caenorhabditis elegans. Bio protocol.

